# The Impact and Determinants of Mountainous Topographical Factors on Soil Microbial Community Characteristics

**DOI:** 10.3390/microorganisms11122878

**Published:** 2023-11-28

**Authors:** Jiantao Yu, Suyan Li, Xiangyang Sun, Wenzhi Zhou, Libing He, Guanyu Zhao, Zhe Chen, Xueting Bai, Jinshuo Zhang

**Affiliations:** The Key Laboratory for Silviculture and Conservation of Ministry of Education, College of Forestry, Beijing Forestry University, Beijing 100083, China

**Keywords:** microbial community, diversity, soil characteristics, elevation, illumina MiSeq sequencing

## Abstract

Soil bacterial and fungal community communities play significant ecological functions in mountain ecosystems. However, it is not clear how topographic factors and soil physicochemical properties influence changes in microbial community structure and diversity. This study aims to investigate how altitude and slope orientation affect soil physicochemical properties, soil microbial communities, and their contributing factors. The assessment was conducted using Illumina MiSeq sequencing in various altitude gradients and on slopes with different aspects (shady slopes and sunny slopes) in the subalpine meadow of Dongling Mountain, Beijing. Topographical factors had a significant effect on soil physicochemical properties: the primary factors determining the structure of microbial communities are total potassium (TK), ammonium nitrogen (NH_4_^+^-N), and soil organic carbon (SOC). There was no significant change in the diversity of the bacterial community, whereas the diversity of the fungal community displayed a single-peaked trend. The effect of slope orientation on microbial communities was not as significant as the effect of elevation on them. The number of bacterial communities with significant differences showed a unimodal trend, while the number of fungal communities showed a decreasing trend. The co-occurrence network of fungal communities exhibits greater intricacy than that of bacterial communities, and bacterial communities are more complex in soils with sunny slopes compared to soils with shady slopes, and the opposite is true for fungal communities. The identification of the main factors that control soil microbial diversity and composition in this study, provided the groundwork for investigating the soil microbial response and adaptation to environmental changes in subalpine meadows.

## 1. Introduction

Mountain ecosystems are one of the typical fragile ecosystems on earth, and the differences in topography and climatic environments make them an open natural platform for studying microbial communities. Subalpine meadow is a special kind of alpine meadow, which is extremely sensitive to the environment, and plays a decisive role in maintaining ecosystem stability and durability [1]. Soil microorganisms are essential constituents of soil ecology, with imperative roles in the material cycle, and the flow of energy within ecosystems [2]. Soil microorganisms are sensible indicators of community composition, ecosystem functioning, and abundance can indicate soil quality, while soil system stability can be reflected by the diversity and structure of soil microorganisms [3]. The soil contains a diverse range of microorganisms, with bacteria and fungi being the most abundant [4]. Studies have shown that in alpine meadows the bacterial community is mainly composed of Proteobacteria and Acidobacteria, and the most numerous fungal communities are Ascomycota and Basidiomycota, and not only with high plasticity and adaptability to the environment, but also, perform a vital function in crucial processes such as plant production, greenhouse gas emissions, and nutrient cycling, which are essential for the maintenance of numerous ecosystem functions [5,6,7,8].

Environmental heterogeneity is seen as a universal driver of microbial diversity [9], where topographic factors lead to dramatic changes in temperature and precipitation indirectly affecting changes in plant traits, soil physicochemical properties, and ecosystem functioning [10], thus driving diversity and variability in the distribution of soil microbial communities [11,12]. Studies exploring forest soil microbial diversity patterns across environmental gradients in montane ecosystems are relatively well-studied, however, there are relatively few studies on the combined effects of topographic factors and soil nutrients on soil microbial communities in subalpine meadows. Slope orientation and elevation are the main topographic parameters that generate environmental heterogeneity in microclimate, pedogenic processes, and vegetation patterns, as well as soil microbial variability. Changes in net solar radiation due to slope orientation lead to substantial soil temperature variations [13,14,15], leading to different soil formations and development of microenvironments, affecting vegetation establishment and there was a decisive impact on the composition of the soil fungal and bacterial community [16]. Consequently, studying fungal and bacterial communities across altitudinal gradients and slope orientations can help to unravel the reply from soil fungal and bacterial to climate change, explore the linkages between topography and microbial communities, and provide insights into their intricate distribution patterns and ecological importance [17,18,19].

The subalpine meadows of Dongling Mountain are located at the edge of the forest and play a crucial role in maintaining the balance and stability of the Dongling Mountain ecosystem. [20]. We selected Dongling Mountain subalpine meadow as the study area to investigate the structural composition and diversity of soil bacterial and fungal communities in meadows with different slope orientations along the altitudinal gradient (shady and sunny slopes) and the main environmental factors influencing the changes have been analyzed as well as the network of microbial communities in different slope orientations. The purpose of this study is to enhance comprehension of the impact of topography and soil factors on soil microbial communities in meadow ecosystems and to promote ecosystem management protection, and restoration. This study will generate data that supports fundamental ecological predictions on the diversity and constituents of soil bacterial communities and soil fungal communities in subalpine meadow ecosystems, in response to climate change.

## 2. Materials and Methods

### 2.1. Description of the Site and the Methodology of the Experiment

Dongling Mountain (39°48′–40°01′ N,115°24′–115°35′ E) is located in Beijing, China, belonging to the remnants of Xiaowutai Mountain in the Taihang Mountain Range, which is the natural ecological environmental protection barrier of Beijing. The highest point of Dongling Mountain is 2303 m above sea level, making it the highest peak in Beijing. It has four distinct seasons, with wet and winter being cold and dry and summer being warm. The average yearly temperature is 5.4 °C, with the highest and lowest temperatures occurring in July and February, respectively. The annual precipitation is 570.3 ± 112.2 mm, with June–August being the peak season every year. The predominant soil types are mountain brown soil and subalpine meadow soil, and their thickness ranges from 90 to 120 cm.

We selected the subalpine meadows with an altitude of 1600~2300 m as the study area in Dongling Mountain Nature Reserve, which had similar slopes and was all free from human interference and selected three altitudinal gradients (1700 m, 1900 m, 2100 m), set two different slope orientations (sunny and shady slopes) at the same altitude, total 6 study areas. And selected three areas of 10 × 10 m were to be sampled using the five-point method, respectively, each of which was more than 100 m apart. Each sampling area was spaced more than 100 m apart, totaling 18 sampling areas (Figure S1). After documenting vegetation information in each sampling area, soil samples were taken from the top 0–20 cm. All samples were collected in sterile plastic bags, placed on dry ice immediately after collection, and transported to the laboratory where they were divided into three portions according to the needs of the study; one portion was stored at −80 °C for DNA high-throughput sequencing analysis, another portion was airdried for physicochemical indexes testing and analysis, and the last portion, collected with a loop knife, was used for investigating and evaluating soil BD and soil moisture(Table 1).

### 2.2. Physical and Chemical Properties of Soil

The soil’s physical and chemical properties were determined following the guidelines provided in the Soil Analysis Handbook (Springer (Berlin/Heidelberg, Germany), 2006). A digital pH meter (1:2.5 *w*/*v*) was used in a soil water sample to determine the pH of the soil. Soil Bulk Density (BD) and moisture content were measured by the drying method, meaning that they were calculated by comparing the weights of soil samples before and after drying in an oven at 105 degrees Celsius for 24 h. Soil-available phosphorus (AP) was determined by the molybdenum antimony anti-colorimetric method with 0.5 M NaHCO_3_. Soil organic carbon (SOC) content was measured using a total organic carbon (TOC) analyzer (Trace Elemental Instruments (Delft, The Netherlands)—XPERT-TOC/TN_b_) Soil’s total nitrogen (TN) content was analyzed via the Kjeldahl method (Shanghai Shengsheng Automation Analysis Instrument Co. (Shanghai, China)—K5100). Total phosphorus (TP) and potassium (TK) levels were determined using the NaOH melt method. The available potassium (AK) in the soil was measured through ammonium acetate-flame photometry (Shanghai Jingke Industry Co. (Shanghai, China)—FP6140). Extraction distillation was used to measure ammonium nitrogen (NH_4_^+^-N), while reduction distillation was employed to measure nitrate nitrogen (NO_3_^−^-N).

### 2.3. The Extraction of DNA and Its Amplification through PCR

Total microbial genomic DNA was extracted from soil samples using the E.Z.N.A.^®^ soil DNA Kit (Omega Bio-tek, Norcross, GA, USA) according to the manufacturer’s instructions. The quality and concentration of DNA were determined by 1.0% agarose gel electrophoresis and a NanoDrop2000 spectrophotometer (Thermo Scientific, Waltham, MA, USA) and kept at −80 °C before further use. The hypervariable region V3-V4 of the bacterial 16S rRNA gene was amplified with primer pairs 338F (5′-ACTCCTACGGGAGGCAGCAG-3′) and 806R (5′-GGACTACHVGGGTWTCTAAT-3′) byT100 Thermal Cycler PCR thermocycler (BIO-RAD, Hercules, CA, USA). The fungal ITS rRNA gene was amplified on a T100 Thermal Cycler PCR thermal cycler (BIO-RAD, Hercules, CA, USA) using primer pairs ITS1F (5′-CTTGGTCATTTAGAGAGGAAGTAA-3′) and ITS2R (5′-GCTGCGTTCTTCATCGATGC-3′). The PCR reaction mixture including 4 μL 5 × Fast Pfu buffer, 2 μL 2.5 mM dNTPs, 0.8 μL each primer (5 μM), 0.4 μL Fast Pfu polymerase, 10 ng of template DNA, and ddH2O to a final volume of 20 µL. PCR amplification cycling conditions were as follows: initial denaturation at 95 °C for 3 min, followed by 27 and 35 cycles of denaturing at 95 °C for 30 s, annealing at 55 °C for 30 s, and extension at 72 °C for 45 s, and single extension at 72 °C for 10 min, and end at 4 °C. The PCR product was extracted from 2% agarose gel and purified using the PCR Clean-Up Kit (YuHua, Shanghai, China) according to the manufacturer’s instructions and quantified using Qubit 4.0 (Thermo Fisher Scientific, Waltham, MA, USA).

### 2.4. Sequencing Data Processing

Purified amplicons were pooled in equimolar amounts and paired-end sequenced on an Illumina PE300 platform (Illumina, San Diego, CA, USA) according to the standard protocols by Majorbio Bio-Pharm Technology Co., Ltd. (Shanghai, China).

### 2.5. Data Analysis

To analyze the soil’s chemical properties and physical properties, as well as the diversity values of soil samples with varying slope directions at different elevations, and to test for statistical significance (*p* < 0.05) using TWO-WAY ANOVA analysis of variance, sorting of data was done using Excel and statistical analysis was carried out using SPSS 27 software. Homogenization of a minimum number of sequences for each soil sample was carried out to minimize the impact of varying sample counts during the analysis of relevant data (34,425 bacterial sequences and 31,691 fungal sequences). Map the microbial communities using R language tools, community Bar mapping was employed, and Circos-0.67–7 was used to plot Circos samples against species. For data analysis of Illumina MiSeq sequencing data, Qiime1.9.1 can generate abundance tables for each taxonomic level and perform beta diversity distance calculations. Mothur1.30.2 can perform alpha diversity analyses to obtain Sobs, Shannon PD, Chao1, and Faith indices. The plotting operation was executed using Origin 2023 software. Normality and variance alignment of the data were assessed using the Levene tests and Shapiro-Wilk, respectively. Beta diversity-based nonmetric multidimensional scaling analysis (NMDS) was conducted to assess bacterial and fungal community similarity, using the Bray-Curtis distance metric. ANOSIM and PERMANOVA were performed using LEfSe for all-to-all comparison (more stringent). We used Redundancy Analysis (RDA) to determine the primary factors influencing the spatial distribution of bacteria and fungi across various gradients and elevations. We calculated the correlation coefficients between environmental factors and selected species using R (version 3.3.1). The resulting matrix was visualized with Heatmap plots.

Analysis of co-occurrence networks was performed using an absolute abundance of dominant OTUs using Networkx 2.8 software. Based on Spearman’s correlation analysis, fungal and bacterial community complexity networks with different slope directions at different elevations were constructed and visualized by Gephi v.0.9.2 and Cytoscaoe v.8.2.

## 3. Results

### 3.1. Physicochemical Properties of Soils at Different Altitude Gradients

Soil pH, soil BD, TN, TP, and NH_4_^+^-N (*p* < 0.05, Table S1) were significantly affected by altitude and had a very significant effect on SOC, soil moisture, TK, and AK (*p* < 0.001). pH showed a significant trend of decreasing with increasing altitude, BD exhibited a pattern of initial decrease followed by an increase, and the Soil moisture, SOC, AK, and NH_4_^+^-N content exhibited an initial increase followed by a decrease. Soil TN and TP were highest at the lowest elevation of 1700 m, and TK was lowest at the lowest elevation of 1700 m. Slope orientation had a conspicuous positive influence (*p* < 0.05) on BD, TK, and AP, and a highly conspicuous positive influence (*p* < 0.001) on Soil moisture and AK. Soil BD and AK were significantly greater on the sunny slope than on the shady side of the north slope, with Soil moisture being greater on the shady than on the sunny side of the slope. TK was greater on the sunny side of the slope than on the shady side, except at 1900 m. It was higher on the shady side of the slope at both 1700 m and 2100 m, while AP was greater on the shady than on the sunny side of the slope at 1900 m, and vice versa at the rest of the elevation. The interaction of slope orientation and elevation had a significant impact on TK and TP (*p* < 0.05) and a highly significant effect on TK, AP, and AK (*p* < 0.001).

### 3.2. Characterization of Soil Microbial Communities

The 16S rRNA genes of soil bacteria and the ITS genes of fungi were sequenced on the Illumina MiSeq platform, and the resulting data were pumped flat to obtain a total of 619,650 optimized soil bacterial sequences and 570,438 optimized soil fungal sequences, of which the valid sequences (removing chimeras) were 355,374 soil bacterial sequences and 555,678 soil Fungal sequences. The average sequence length distributions of soil bacteria and fungi were 418 bp and 246 bp, and their 16S and ITS coverage indices were >96% and >99%, respectively. The gene dilution curves of all samples leveled off at 97% OUT sequence similarity (Figure S2), the information that was gathered through this sequencing was sufficient for analyzing the composition and diversity of the bacterial and fungal communities present in every sample.

**Figure 1:**
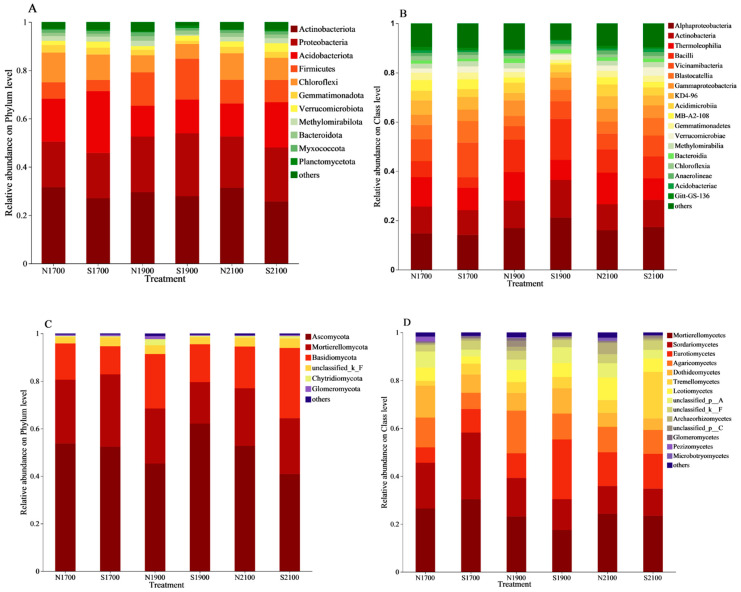
Comparison of the relative abundances of the major Bacterial phyla (**A**) and classes (**B**) found in soil. Fungal phyla (**C**) and classes (**D**) for a variety of slope directions and soils at several different elevations. N for north, shady slopes, S for south, sunny slopes, for example, N1700 means 1700 m above sea level on the north slope.

A total of 3391 soil bacterial OTUs, distributed across 35 phyla, 105 classes, and 611 genera were identified. Actinobacteriota (28.84% of the total OTUs), Proteobacteria (21.74%), and Acidobacteriota (17.08%) accounted for 67.66% of the total 67.66%. Proteobacteria (*p* < 0.05) were significantly affected by altitude, exhibiting an increasing and then decreasing trend, and slope orientation had a greater significant influence on Actinobacteriota (*p* < 0.01) being more shaded than sunny (Figure 1A, Table S2). The relative abundance of Alphaproteobacteria, Actinobacteria, and Thermoleophilia at the class level was 16.70%, 11.53%, and 10.38%, respectively, and together accounted for 38.61% of the total, being the dominant phyla (Figure 1B). Altitude had a conspicuous influence (*p* < 0.05) on Alphaproteobacteria and Actinobacteria, both showing an increasing and then decreasing trend. Slope orientation had a conspicuous effect (*p* < 0.05) on Thermoleophilia, there was a significant increase in shady slopes compared to sunny slopes. Actinobacteria (*p* < 0.05, Table S2) Actinobacteria were significantly influenced by the interplay of altitude and slope orientation. Soil bacterial community at genus level Bacillus (6.82%), Vicinamibacterales (5.89%), and RB41 (4.56%) were the dominant genera accounting for 17.27% of the total bacterial sequences.

A total of 3994 soil fungi OTUs were identified, which were distributed among 15 phyla, 59 classes, and 673 genera. Among them, Ascomycota, Mortierellomycota, and Basidiomycota were the dominating phyla, accounting for 51.19%, 24.28%, and 18.80%, respectively, of the total quantity of obtained bacterial sequences, totaling 94.27%, while the remaining 12 phyla accounted for only 5.73% (Figure 1C). The interaction of elevation and slope orientation had a significant influence (*p* < 0.05) on Ascomycota. The relative abundance of Mortierellomycetes, Sordariomycetes, and Eurotiomycetes at the class level was 24.22%, 16.46%, and 13.37%, respectively, and together they accounted for 54.05% of the total, making them dominant phyla (Figure 1D). Altitude had a highly significant effect on Sordariomycetes (*p* < 0.001), which tended to decrease with increasing altitude, and both altitude and slope orientation had a significant impact on Eurotiomycetes (*p* < 0.05, Table S3), reaching the highest abundance at 1900 m on the sunny slopes, and the interaction between slope orientation and altitude had a major influence on the relative abundance of Sordariomycetes, and Eurotiomycetes had a significant influence (*p* < 0.05). The dominant genera in the soil fungal community were Mortierella (24.26%), Exophiala (5.38%), and Ascomycota (5.02%), which accounted for 34.66% of the total number of fungal sequences.

**Figure 2:**
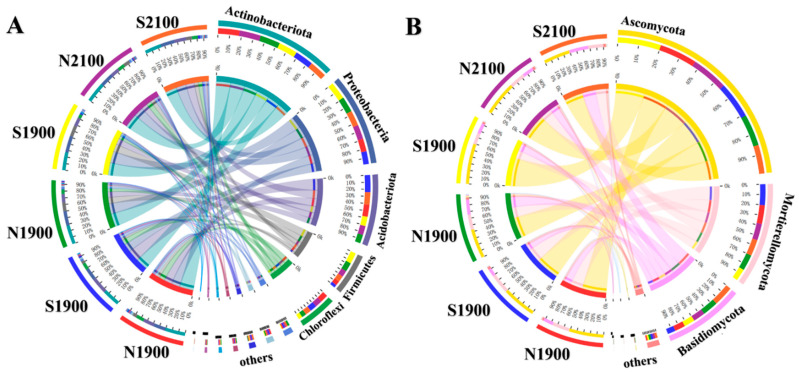
Relative abundances of dominant Bacterial phylum (**A**) and Fungi phylum (**B**) in the soil under three altitude gradients and two slope aspects. The width of the bars from each phylum indicates the relative abundance of that phylum in the sample.

The dominant flora was similar in the different slope orientations at different altitudes, and the abundance of soil bacterial Actinobacteriota was greater than that in the shaded and sunny slopes of the three altitudinal gradients of 1700 m, 1900 m, and 2100 m in a total of six samples, increasing by 11% in the shaded and sunny slopes at each altitudinal gradient. In terms of the whole collection of Actinobacteriota species, the proportion of shaded slopes was also greater than that of sunny slopes for all three elevation gradients, with a total increase of 6%. For the six samples in this study, the proportion of Proteobacteria abundance increased by 11% on the 1900 m gradient compared to the 1700 m gradient, and for the total number of Proteobacteria species, the 1900 m gradient was also higher than the 1700 m gradient, with an increase of 10% (Figure 2A). For Ascomycota, the dominant soil fungal phylum, the highest percentage of Ascomycota abundance, S1900, was 21% higher than the lowest percentage of abundance, S2100, in the six samples. Of the total Ascomycota species, S1900 was 7% higher than S2100 (Figure 2B).

### 3.3. Index of Soil Microbial Community Diversity

**Figure 3:**
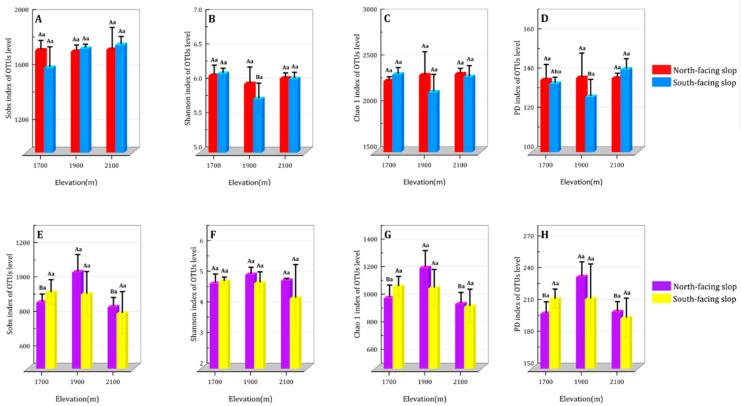
Sobs, PD, Shannon, Chao1 indices of bacterial (**A**–**D**) and fungal (**E**–**H**) communities at different altitudes and slops. Differences between shaded and sunny slopes are written in uppercase (*p* < 0.05), and differences across the three elevation gradients are written in lowercase (*p* < 0.05).

Altitude significantly affected both the Shannon and PD diversity indices of the soil bacterial community, displaying a decreasing and then increasing trend. The Shannon index of soil bacteria on sunny slopes at 1900 m was 6.4% lower than that at 1700 m (*p* < 0.05), and the PD index of soil bacteria at 1900 m was 10.9% lower than that at 2100 m (*p* < 0.05). The orientation of the slope did not significantly impact the alpha diversity of soil bacterial communities (Figure 3A–D). The altitude significantly influenced the sobs, Chao 1, and PD diversity indices of soil fungal communities. The total fungal community diversity showed a unimodal pattern of increasing and then decreasing. Moreover, on shaded slopes, fungal sobs and Chao 1 diversity indices were 23.7% and 27.1% higher, respectively, at 1900 m than at 2100 m (*p* < 0.05). At 1900 m, the fungal PD diversity index was 17.1% higher than that at 1700 m (*p* < 0.05). There was no discernible effect that could be attributed to the direction of the slope on the alpha diversity of the soil fungus communities (Figure 3E–H).

**Figure 4:**
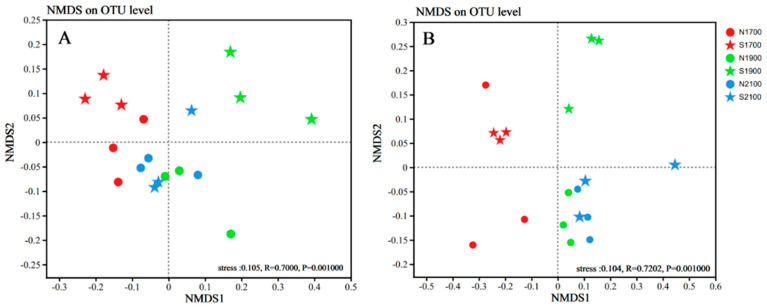
NMDS analyses were conducted on the soil bacterial (**A**), and fungal (**B**) communities.

The NMDS analyses were performed on soil fungal and bacterial sequencing data collected from different altitudes exhibiting varied slope orientations, based on Bray–Curtis distance and both bacterial and fungal NMDS analyses had STRESS values less than 0.2, indicating that they were representative. The grouping of soil bacterial and soil fungal communities was partially influenced by altitude, and the bacterial and fungal aggregation was firmer and more similar on the 2100 m. It was evident that the bacterial communities and fungal communities at 1700 m and 1900 m, were organized according to slope direction. (Figure 4A,B). According to the ANOSIM and PERMANOVA analyses, there is a significant difference (*p* < 0.01) between the fungal and bacterial community structures at different altitudes. The PERMANOVA analysis of all the samples revealed that altitude had a more demonstrable impact on the structure of the fungal and bacterial communities in the soil than slope. (*p* < 0.01, Table 2).

**Figure 5:**
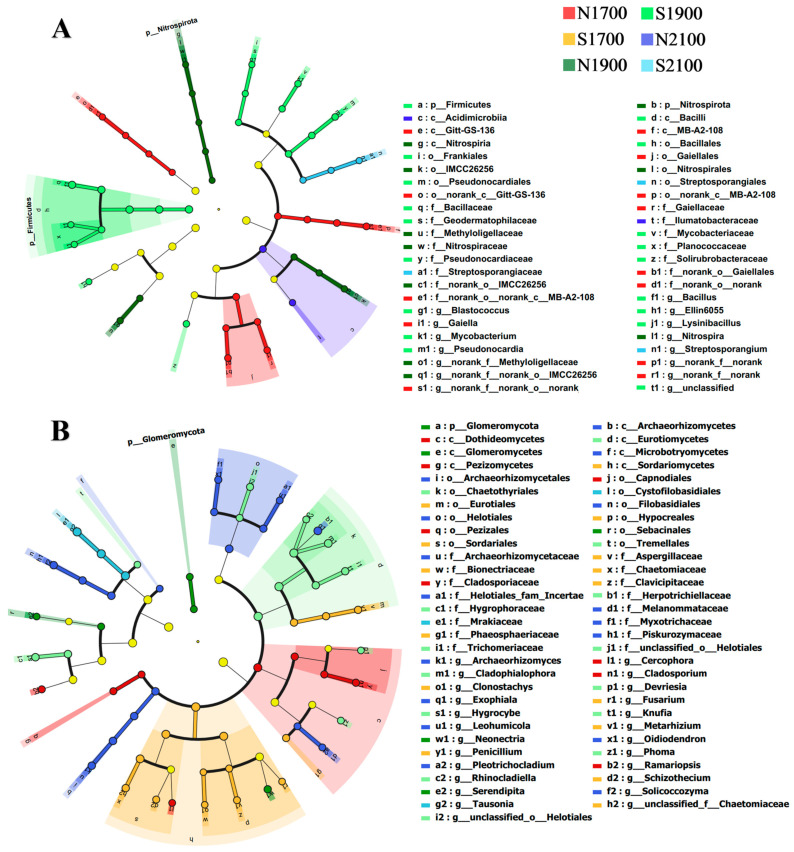
Variations in the community structure of soil bacteria (**A**) and fungi (**B**) at various altitudes and slope aspects in subalpine meadows. Different-colored regions represent different study areas. Circles indicate phylogenetic levels from phylum to genus. The diameter of each circle is proportional to the abundance of the group.

Discriminant analyses of different taxonomic levels (from phylum to genus) of soil microorganisms at different altitudes and slope orientations using LEfSe analysis revealed statistically significant differences in community types for bacteria and fungi. When the LDA threshold was 3.5, a total of 46 bacterial branches showed statistical differences (*p* < 0.05, Figure 5A) in the bacterial community at different altitudes and slope orientations and different taxonomic levels, and 62 branches in the fungal community showed statistical differences (*p* < 0.05, Figure 5B). At various altitudes and slope orientations, the number of soil bacterial and fungal communities exhibited significant variation. As the elevation increased, the number of bacterial communities with significant differences exhibited a monotonic increase and then decrease. At a gradient of 1700 m, the slopes that are shaded are higher than the slopes, with no significant differences in bacterial branches on the sunny slopes at the 1700 m elevation gradient, and higher on sunny slopes than on shady slopes, and higher on sunny slopes than on the shady slopes at the 1900 m and 2100 m elevation gradients (Figure S3A), with only three bacterial branches significantly enriched on the sunny slopes at the 2100 m elevation gradient and only two bacterial branches significantly enriched on the shaded slope. The number of fungal communities with significant differences with increasing elevation showed a decreasing trend, and the number of fungi on the sunny slopes was greater than that on the shady slopes, except for the 2100 m elevation gradient (Figure S3B), where only three fungal branches were significantly enriched on the sunny slopes of the 2100 m elevation gradient.

### 3.4. Association between Soil Physicochemical Properties and Soil Microbial Communities

**Figure 6:**
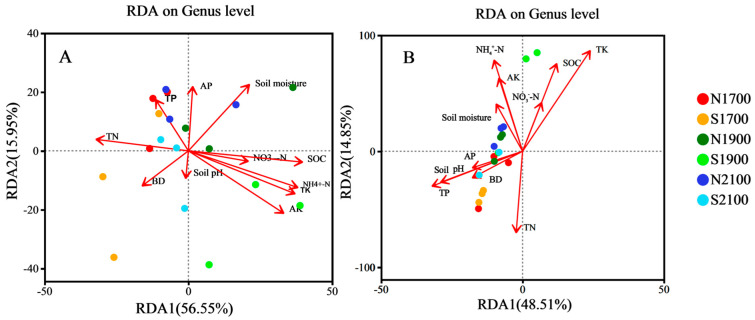
Analysis of soil bacterial genus (**A**), fungal genus (**B**) communities, and factors (red arrows) for Redundancy. BD: Soil bulk density; TN: Total nitrogen; TP: total phosphorus; AK: Available potassium; SOC: soil organic carbon; TK: total potassium AP: Available phosphorus; NO_3_^−^-N: Nitrate nitrogen; NH_4_^+^-N: Ammonium nitrogen.

The article analyzes redundancy and correlation to reflect and evaluate the impact of soil environmental factors on the structure of microbial communities, while also assessing their correlation using heatmap plot analysis. According to the genus-level bacterial community RDA plot, the first two axes of the soil bacterial community explain over 72% of the structural changes (Figure 6A), which was biostatistically significant. Among them, NH_4_^+^-N, TK, AK, and SOC had a significant effect (*p* < 0.01) with R2 of 0.644, 0.639, 0.63, and 0.629, respectively, followed by TN and soil moisture (*p* < 0.05). For soil fungi, as shown, more than 63% of the structural variation was explained by the first two axes (Figure 6B), which was biologically statistically significant. Among them, TK, NH_4_^+^-N, SOC, and TN produced highly significant effects (*p* < 0.01) with R2 of 0.689, 0.511, 0.489, and 0.404, followed by AK (*p* < 0.05).

**Figure 7:**
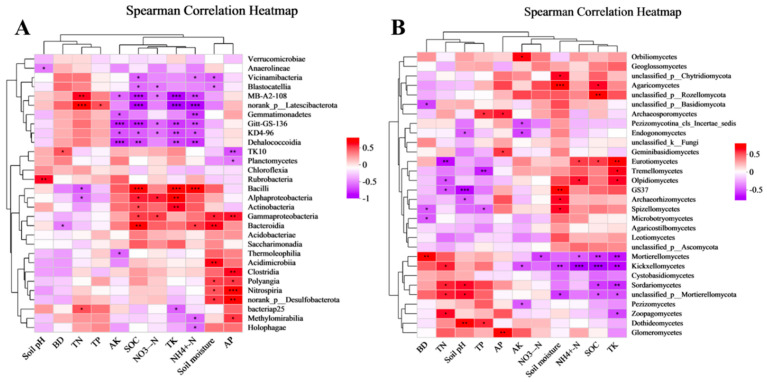
Heatmap depicting the associations between bacterial (**A**) and fungal (**B**) communities and soil variables. BD: Soil bulk density; TN: Total nitrogen; TP: total phosphorus; AK: Available potassium; SOC: soil organic carbon; TK: total potassium AP: Available phosphorus; NO_3_^−^-N: Nitrate nitrogen; NH_4_^+^-N: Ammonium nitrogen. *, **, *** indicate statistical significance at *p* < 0.05, *p* < 0.01, *p* < 0.001.

According to the compartmentalized soil bacterial communities and fungal communities heatmap (Figure 7A,B), the same environmental factor had different effects on different microbial communities, and in the soil bacterial community, SOC, TK, and NH_4_^+^-N had highly significant positive correlation (*p* < 0.001) and highly significant negative correlation (*p* < 0.001) correlations with the different bacterial communities, respectively. (*p* < 0.001). Based on their clustering, microbiological communities in the soil can be observed, soil pH, BD, TN, and TP had similar effects on bacteria and fungi, and NH_4_^+^-N, NO_3_^−^-N, AK, TK, and SOC had a similar influence on bacteria and fungi. Based on the clustering of the bacterial communities and fungal communities, the various responses to environmental factors at the phylum level were categorized into four groups of bacterial communities and four groups of fungal communities.

### 3.5. Co-Occurrence Network of Soil Microbial Communities in Different Slope Aspects

**Figure 8:**
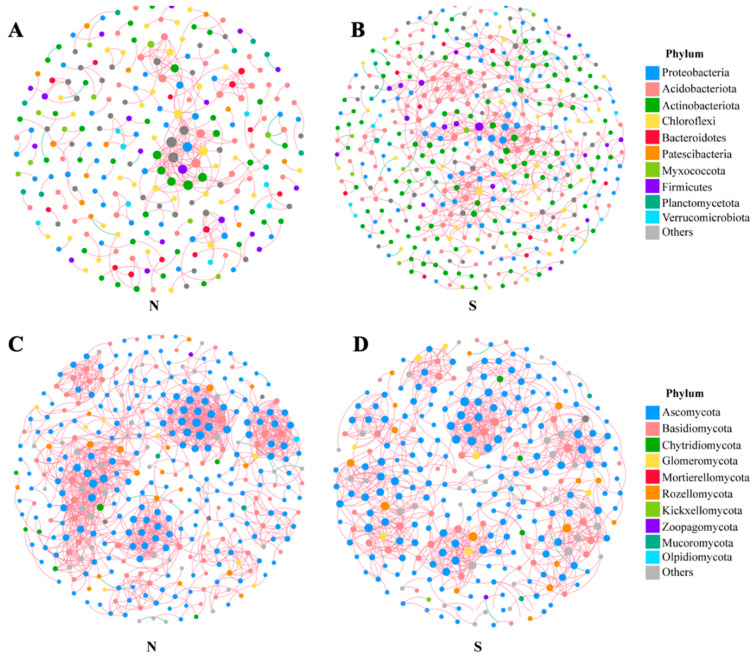
Network co-occurrence analysis of bacteria in the shaded slope (**A**) and fungi in the shaded slope (**B**), and bacteria in the sunny slope (**C**) and fungi in the sunny slope (**D**).

In the four co-occurrence networks (Figure 8). The shaded slope bacterial community had 312 links (edges) out of 262 OTUs (nodes), of which 302 (96.79%) were positively correlated and 10 (3.21%) were negatively correlated. The sunny slope bacterial community has 663 links (edges) out of 427 OTUs (nodes), of which 643 (96.98%) are positively correlated and 20 (3.02%) are negatively correlated. The shaded slope fungal community has 1333 links (edges) out of 359 OTUs (nodes), of which 1326 (99.47%) are positively correlated and 7 (0.53%) are negatively correlated. The sunny slope fungal community has 990 links (edges) out of 289 OTUs (nodes), of which 983 (99.29%) are positively correlated and 7 (0.71%) are negatively correlated.

## 4. Discussion

### 4.1. Alterations in Physicochemical Properties of Soil along Altitudinal Gradients and Slope Aspects

In this study, there was an increase and subsequent decrease in soil moisture, SOC, TK, AK, NO_3_^−^-N, and NH_4_^+^-N content as altitude increased. This may be due to vegetation community richness. It is interesting that plant richness also demonstrated the same tendency, which is an increase followed by a decrease with rising altitude. The highest number of plant species was observed at an altitude of 1900 m. Zhou’s (2019) research demonstrated that altitude significantly affects plant growth and distribution in subalpine meadows. Hydrothermal conditions vary with altitude and slope direction, leading to an uneven distribution of the environment, causing differences in the composition of plant communities [21], further leading to the uneven distribution of plant communities and changing the nutrient content of the soil. As the altitude increased, grassland vegetation richness and cover at 1900 m reached a maximum, apoplastic material increased, and air temperature and potential evapotranspiration decreased. As a result, the soil moisture content increased and the soil BD decreased. This increased grassroot vigor and productivity, causing root necrosis and secretions that added more carbon and other elements to the soil [13]. This led to a positive feedback cycle that increased some of the soil’s nutrient contents. With increasing altitude up to 2100 m and reaching the summit of the main peak of Dongling Mountain, the ambient temperature decreased further. The low temperature was unfavorable for plant growth and led to a decrease in vegetation richness. Evaporation of soil increased, causing a decrease in water content and an increase in soil capacity [22]. This led to a decrease in some soil nutrient contents. Furthermore, at the same altitude in our study, soil moisture, SOC, TP, TN, TK, and AK were greater than or equal to those on sunny slopes at the same altitude. This could be attributed to the significant difference in solar radiation at the same altitude on shady slopes when compared to sunny slopes. It is the main determining factor for soil water evaporation, temperature, carbon, and nitrogen cycling, resulting in significant differences in soil moisture content and bulk density [13].

### 4.2. Community Composition and Diversity of Soil Microbial Communities

#### 4.2.1. Structural Composition of Soil Microbial Communities at Different Altitudes and Slope Aspects

The microbial abundance of different taxa at different taxonomic levels responded differently to elevation and slope direction, which requires further investigation at finer taxonomic levels. In our study, we found that the dominant phyla of soil bacterial communities were Actinobacteriota, Proteobacteria, and Acidobacteriota, which is in keeping with previous research indicating that these microbes are prevalent in the soils of various ecosystems [23]. Changes in the three altitudinal gradients have a strong influence on the bacterial phylum, which is relatively abundant in soil microorganisms (Proteobacteria, Firmicutes, Chloroflexi, and Gemmatimonadota) and slope orientation had a significant effect on Actinobacteriota and Thermoleophilia in the bacterial community. He’s (2023) showed that altitudinal gradients are associated with changes in different environmental factors that trigger different habitat-specific patterns and have different effects on different microbial communities [24,25,26]. Zhou’s (2019) showed that Actinobacteriota, an oligotrophic taxon with strong metabolism and DNA repair mechanisms even at low temperatures, prefers nutrient-poor alkaline environments [21,27]. This is consistent with our study that the total percentage of Actinobacteriota was the highest in the study area and the relative abundance of Actinobacteriota was the lowest in the area of the 2100 m elevation gradient on the sunny slopes with the lowest pH. Proteobacteria were mainly affected by the significant positive correlation between NO_3_^−^-N, TK, SOC, and NH_4_^+^-N, which showed off the three altitude gradients, the lowest altitude gradient had the lowest levels, which then increased to the highest point before decreasing. Showing a single peak pattern, which is consistent with the previous view that Proteobacteria prefer eutrophic conditions [28]. Proteobacteria, although involved in energy metabolism and playing key roles in phylogenetic, ecological, and pathogenic values, have a symbiotic lifestyle, and are less abundant in harsh and infertile soil environments, which explains why from 1900 m to 2100 m Proteobacteria the relative abundance of Acidobacteriota decreased from 1900 m to 2100 m. Acidobacteriota had a significant negative correlation with soil moisture, NO_3_^−^-N, SOC, and NH_4_^+^-N. Acidobacteriota plays a significant role in the decomposition of organic matter and nutrient cycling [29].

Ascomycota, Mortierellomycota, and Basidiomycota were the dominant phyla of the soil fungal communities, and the results of this research were comparable to those of Li’s (2022) study, and Chen’s (2022) study [22,30]. Changes in the three altitudinal gradients in this study had a significant influence on fungal phyla (Sordariomycetes and Eurotiomycetes). Slope direction had a significant effect on Eurotiomycetes in the fungal community. The greatest abundance of Ascomycota in the subalpine meadows of Dongling Mountain can be mainly attributed to its strong reproductive ability, rapid growth, a quantitative advantage in producing a large number of spores, and the fact that Ascomycota is more adaptable to harsh habitats. Mortierellomycota had the highest abundance at an altitude of 2100 m. Fungal saprophytes such as Mortierellomycota and Mucor. are considered high-altitude opportunists due to their tolerance to low temperatures and may be able to colonize and survive on high-altitude peaks [31]. Basidiomycota fungi have a potent capacity for lignocellulose decomposition, and in contrast, in this study, the relative abundance of Basidiomycota in subalpine meadow soils decreased with increasing elevation gradients, possibly because increased nutrient efficiency inhibits its growth [4].

#### 4.2.2. Soil Microbial Community Diversity at Different Altitudes and Slope Aspects

Soil microbial diversity is a direct reflection of changes in its abundance, richness, and uniformity, and the majority of previous studies reporting the pattern of soil microbial diversity at various altitudes using high-throughput sequencing techniques have yielded comparable results. The study revealed a decrease in the diversity of soil fungal and bacterial communities with increasing altitude [32,33,34]. This partially supports the conclusion that the diversity and structural composition of soil bacterial and fungal communities decrease with an increase in altitude. Generally, the environmental conditions became harsher as the altitude increased, and it was therefore predicted that the abundance of soil microbes and fungi would decrease along the gradient of altitude [35]. Our study found that soil bacterial diversity in subalpine meadows did not exhibit a clear trend with increasing elevation. Instead, it showed a general trend of decreasing until reaching the lowest point and then increasing. This is not entirely consistent with the findings of previous studies. However, consistent with the findings of Fierer’s (2011) study that although there was There are no changes that can be seen at a glance in bacterial diversity across the gradient, significantly different compositions of soil bacterial communities in subalpine meadows across three altitudinal gradients [10]. This may be because bacterial community diversity is mainly influenced by soil pH, and there were two altitudinal gradients in the study area where pH was not significantly different [36]. In our study, soil fungal diversity showed a unimodal trend of increasing with altitude and then decreasing after reaching the highest point of elevation. Recent studies have highlighted the inconsistency in the physiological geographical patterns of bacteria and fungi [33]. Distinct vertical distribution patterns of soil bacteria and fungi at altitude arise due to significant differences in the life and evolutionary histories of various taxa. The results of Li’s (2022) are in agreement with the unimodal trend observed in fungal communities [22]. This may be the result of elevated soil nutrient concentrations (soil moisture, SOC, AK, NH_4_^+^-N) at 1900 m, which favored the growth of soil microorganisms. In addition, for the soil bacterial community, the bacterial diversity at three altitudes was relatively flat on the sunny slopes with no significant change, while it showed a decreasing and then increasing trend on the shady slopes. When it came to the fungal community in the soil, the soil fungal diversity index was greater on the sunny side than on the shady side at the same elevation, except at 1700 m. These findings are comparable to those of Li’s (2022), who conducted a study on the same topic [22]. It was found that the diversity index of the meadow soil bacterial community was stronger than that of the meadow soil fungal community. Ji’s (2022) research in the cold temperate zone supports this finding. Additionally, the study proposes that various microbial taxa in Dongling Mountain have evolved niche differentiation across the altitudinal gradient [30].

The response of the features of the grassland vegetation and the soil nutrient variables to slope and elevation was consistent with the variation in the bacterial and fungal diversity indices found in the soil. Soil physicochemical characteristics may affect soil microbial communities [37]. The beta diversity of soil bacterial communities and soil fungal communities could be distinguished visually at different elevations and slope directions, the properties of soil fungal communities vary considerably more with altitude and slope direction than those of soil bacterial communities in our study. Changes in elevation and slope direction typically lead to variations in climatic factors, soil characteristics, and vegetation, resulting in alterations in the composition of bacterial and fungal communities, and increased species richness and diversity, to facilitate adaptation to altered environmental conditions [38].

### 4.3. Environmental Factors Drive Soil Microbial Communities

Previous research has demonstrated that soil organic carbon substantially affects the composition and diversity of soil fungal communities in alpine meadows during succession [34]. Wang’s (2023) showed that the pH of the soil is an essential factor of bacterial community structure in various soil ecosystems, and it indirectly influences soil bacterial habitats by modulating other influences, thereby influencing the composition of soil bacterial communities [36,39,40]. Nevertheless, the results of our study differ from previous studies, we found that soil physicochemical variables had a high degree of explanatory power for bacterial community composition and that NH_4_^+^-N, TK, AK, and SOC had a significant impact on soil bacterial communities, with NH_4_^+^-N having the greatest influence. And TK, TN, NH_4_^+^-N, SOC had a significant impact on the soil fungal community, with TK having the greatest effect. Soil pH in this study differed significantly across the altitudinal gradient, with the high altitude of 2100 m being lower than other altitudinal gradients, but the effects on soil microbial communities were not significant, as can be seen from the heat map graph, which shows that there was only a slight negative correlation with Basidiomycota of the fungal community. The research of Zheng (2019) demonstrated that certain soil bacterial communities could adapt to variations in soil pH [41]. It is believed that SOC, as a basic substrate and energy source for soil microorganisms, and Soil carbon and soil nitrogen greatly influence microbial distribution patterns at elevation and slope by influencing the metabolism of soil microbial communities [16]. In alpine meadows, Previous research has found that the relative abundance of taxa clades (Actinobacterioidae and Firmicutes) varied with soil organic carbon, indicating that soil carbon efficiency influences soil bacterial diversity and soil community composition [17]. Furthermore, soil NH_4_^+^-N strongly influenced microbial diversity and the composition of soil fungal and soil bacterial communities in subalpine meadows, supporting the notion that soil conditions influence Soil microbial diversity and soil microbial community composition. Soil-effective nitrogen represents the process of microbial dynamic nitrogen cycling and increases in nitrogen nutrients have a greater significant impact on the constitute of microbial communities by regulating microbial nitrogen energy sources [42].

Bacterial communities are more affected by altitude and slope as compared to fungal communities, perhaps due to the narrower physiological spectrum of soil fungi. For instance, soil bacteria may be photosynthetic autotrophs or chemoautotrophs, but soil fungi are only heterotrophs [43]. Additionally, we confirmed that the bacterial community in the subalpine meadow ecosystem of Dongling Mountain, which is in the warm temperate zone, was more affected by slope and altitude direction than the fungal community, and that elevation had a greater significant impact on the structure of the microbial communities than slope direction. Changes in elevation and slope factors synergistically influence the composition and diversity of soil bacterial and fungal communities in subalpine meadows. The community structure of soil microbes and fungi can be altered in response to environmental stimuli.

### 4.4. Co-Occurrence Networks of Microbial Communities with Different Characteristics on Shady and Sunny Slopes

Microorganisms in natural ecosystems are not simple assemblages of individual populations, however, these organisms create elaborate ecological networks that are essential for the maintenance of ecosystem processes and services [44]. Co-occurrence networks reveal dominant microorganisms and closely interacting microbial communities that are critical for preserving the stability of the microbial community structure and functioning in the environment [45]. Li’s (2023) showed that along the elevation gradient, the distribution patterns of bacterial and fungal community symbiosis networks differed, possibly due to ecological niche differentiation caused by the high variability of the soil environment and plant communities [46]. We speculated that the structure of the co-occurrence network between soil microbial communities would vary depending on whether the slope was shaded or sunny. We found that all four networks showed significantly greater modularity and clustering coefficients compared to the random network. The co-occurrence networks of the soil fungal communities were more interactive than those of the soil bacterial communities, and the increased complexity indicates greater stability and environmental resistance of the fungal communities. This is in line with our assumption. Co-occurrence networks of the microbial communities on the shaded and sunny slopes changed significantly, but the positive correlation ratios were both much larger than the negative correlation ratios, indicating that both had synergistic effects and that there was more cooperation and reciprocity than competition in the microbial communities [47,48,49]. In the bacterial community, the number of nodes, the number of links, and the positive correlation links were slightly increased in the sunny slope compared to the shaded slope, indicating that the co-occurrence network of the bacterial community was more developed in the sunny slope. The dominant phylum Actinobacteriota, Acidobacteriota, Proteobacteria, and Chloroflexi were more strongly associated with the bacterial community. The total number of nodes, links, and positively correlated links in the fungal community was greater on the shady side of the slope than on the sunny side. Ascomycota were the most significant elements in the fungal community. The distribution and co-occurrence network of fungal and bacterial communities in subalpine meadow ecosystems varied with slope direction.

## 5. Conclusions

Our study showed that altitude and slope orientation play a significant role in the variation of soil physicochemical properties and have a significant effect on the composition and diversity of soil fungal and bacterial communities. Altitude had a significant influence on soil pH, soil bulk density, total nitrogen, total phosphorus and ammonium nitrogen, soil moisture, soil organic carbon, soil total potassium, and available potassium, and slope orientation had a significant influence on soil bulk density, total potassium and available phosphorus, soil moisture, and available potassium. Significant variations were found between the main phyla and genera found in the soil bacterial and fungal communities of subalpine meadows located at different elevations and along different slopes. The bacterial dominant phyla were Actinobacteriota (28.84%), Proteobacteria (21.74%), and Acidobacteriota (17.08%), while the fungal dominant phyla were Ascomycota (51.19%), Mortierellomycota (24.28%) and Basidiomycota (18.80%). An increase in elevation had a significant effect on Shannon and PD diversity indices of soil bacterial communities, showing a decreasing and then increasing trend. The soil fungal community showed a single-peaked trend of increasing and then decreasing with increasing altitude. Except at 1700 m, the diversity of soil fungi was greater on shady slopes than on sunny slopes at the same altitude. Bacterial diversity was greater than fungal diversity overall. The co-occurrence network of communities of fungi was considerably more intricate than that of communities of bacteria, with the bacterial community network being more complex in soils on sunny slopes and the fungal community network being more developed in soils on shady slopes. Soil total potassium, ammonium nitrogen, and soil organic carbon were the primary factors influencing the community structure of bacteria and fungi. Additional research on soil microbial communities in subalpine meadows would allow us to comprehend the dispersion of microbial communities in the various topographies present in this delicate ecosystem. It would also establish a theoretical foundation to anticipate the reflection, adaptations, and feedback of microbial communities’ changes with the environment, which is pivotal in assessing the sustainability of meadow ecosystems and having a comprehensive understanding of microbial ecology.

## Figures and Tables

**Table 1:** Site characteristics of different altitudes.

Altitude (m, above Sea Level.)	Aspect	Coordinates	Soil Type	Dominant Plant Taxa
1700	Sunny	40°2′02″ E, 115°29′38″ N	paramo soil	*Polygonum aviculare* L., *Artemisia carvifolia*, *Sedum aizoon* L., *Vicia cracca* L., *Poa spondylosis Trin.*, *Equisetum arvense* L., *Dendranthema chanetii*, *Bupleurum chinense DC.*, *Halenia corniculata*, *Delphinium grandiflorum*, *Dianthus chinensis* L.
	Shady	40°2′31″ E, 115°29′25″ N		
1900	Sunny	40°2′01″ E, 115°28′56″ N	paramo soil	*Sanguisorba officinalis* L., *Rumex acetosa* L., *Sedum aizoon* L., *Carex* L., *Asparagus cochinchinensis*, *Dendranthema chanetii*, *Heteropappus hispidus* (*Thunb.*), *Delphinium grandiflorum*, *Echinops sphaerocephalus* L., *Allium wallichii*, *Dendranthema zawadskii*, *Dracocephalum rupestre Hance*, *Viola verecunda*, *Artemisia sacrorum Ledeb.*, *Arundinella hirta*, *Saussurea japonica*, *Saussurea Chinensis*, *Dianthus chinensis* L.
	Shady	40°2′21″ E, 115°28′57″ N		
2100	Sunny	40°2′04″ E, 115°28′42″ N	paramo soil	*Carex duriuscula C.*, *Potentilla discolor Bge.*, *Sanguisorba officinalis* L., *Euonymus fortunei*, *Carex* L., *Dendranthema zawadskii*, *Stellaria dichotoma* L., *Gentiana macrophylla Pall.*, *Saussurea japonica*, *Saussurea Chinensis*, *Heteropappus hispidus*, *Ctenopteris*
	Shady	40°2′12″ E, 115°28′41″ N		

**Table 2:** Non-parametric multivariate analysis (PERMANOVA) and ANOSIM soil fungal and bacterial community analysis by altitude and slope aspect.

	ANOSIM		PERMANOVA		
	Statistic	*p*		R^2^	*p*
Bacteria	0.7000	0.001	Altitude	0.60805	0.001
			Slope aspect	0.11409	0.042
Fungi	0.7202	0.001	Altitude	0.58703	0.001
			Slope aspect	0.09881	0.031

## Data Availability

The data presented in this study are available upon request from the corresponding author.

## Supplementary Materials

The following supporting information can be downloaded at: https://www.mdpi.com/article/10.3390/microorganisms11122878/s1, Figure S1: Location of the research area; Figure S2: Rarefaction contours of operational taxonomic units (OTUs) for soil bacterial (A) and fungal (B) communities; Figure S3: Variations in the community structure of soil bacteria (A) and fungi (B) at various altitudes and slope; Table S1: The soil physicochemical properties for South-facing slop and North-facing slop soils in different altitudes; Table S2: Two-way variance analysis of dominant bacterial phyla and classes; Table S3: Two-way variance analysis of dominant fungal phyla and classes.
