# Supplementary material for: The Impact and Determinants of Mountainous Topographical Factors on Soil Microbial Community Characteristics
Source: Microorganisms. 2023 Nov 28;11(12):2878. doi: 10.3390/microorganisms11122878 (PMC10746091; doi:10.3390/microorganisms11122878)
Supplement: Supplementary file 1 [file microorganisms-11-02878-s001.zip › microorganisms-2694856-supplementary.pdf]

Supplementary Material

Supporting Information for the manuscript paper entitled: "***The impact and determinants of mountainous topographical factors on soil microbial community characteristics***" by Jiantao Yu [1], Suyan Li [1,*], Xiangyang Sun [1], Wenzhi Zhou [1], Libing He [1], Guanyu Zhao [1], Zhe Chen [1], Xueting Bai [1], Jinshuo, Zhang [1]

1 The Key Laboratory for Silviculture and Conservation of Ministry of Education, College of Forestry, Beijing Forestry University, Beijing 100083, China.

* Correspondence: lisuyan@bjfu.edu.cn; sunxybjfusoil@163.com

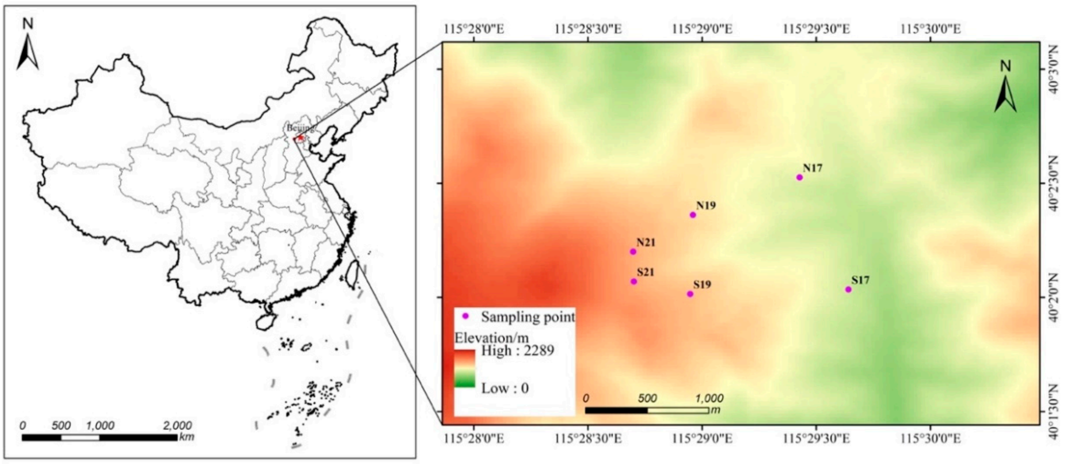

Figure S1. Location of the research area

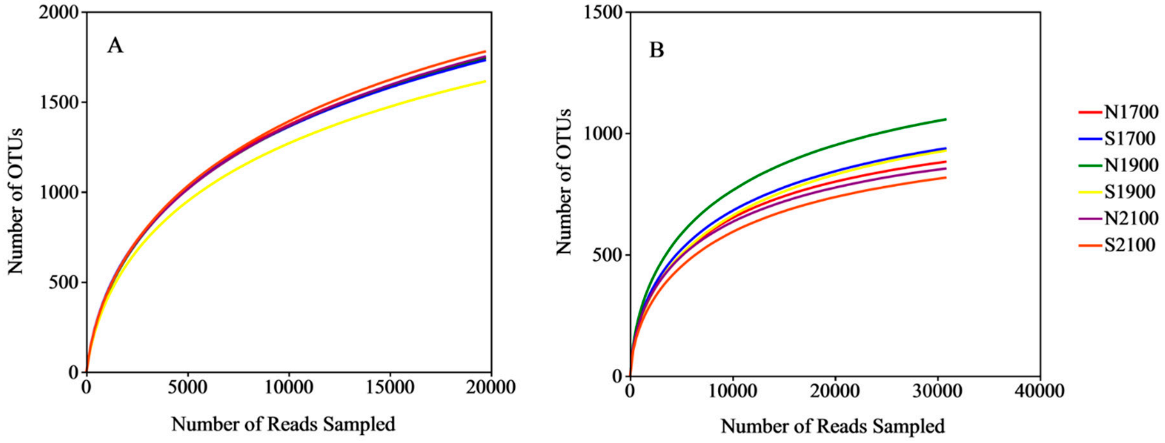

Figure S2. Rarefaction contours of operational taxonomic units (OTUs) for soil bacterial (A) and fungal (B) communities. N for north, shady slopes, S for south, sunny slopes

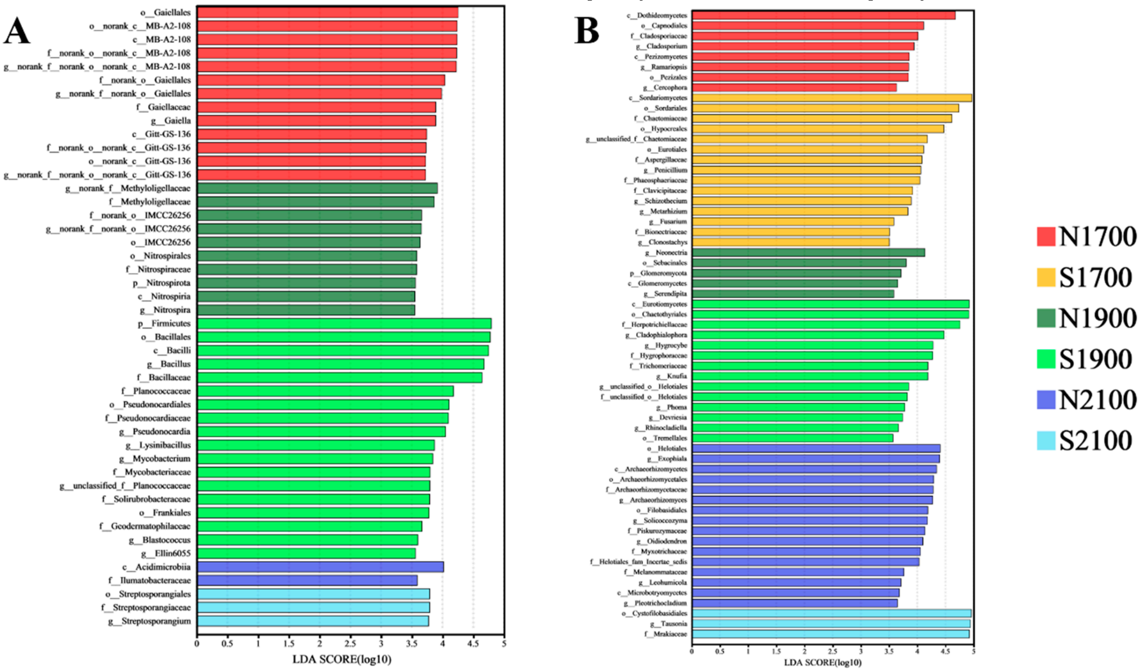

Figure S3. Variations in the community structure of soil bacteria (A) and fungi (B) at various altitudes and slope. N for north, shady slopes, S for south, sunny slopes

Table S1. The soil physicochemical properties for South-facing slop and North-facing slop soils in different altitudes.

		Soil pH	BD (g·cm-3)	Soil moisture (%)	SOC (g·kg-1)	TN (g·kg-1)	TP (g·kg-1)	TK (g·kg-1)	AP (mg·kg-1)	AK (mg·kg-1)	NH_4^+-N (mg·kg-1)	NO_3^--N (mg·kg-1)
**South-facing slop**	S1700	7.21±0.03 Aa	1.13±0.04 Aa	28.24±2.72 Ab	32.52±1.74 Ac	2.40±0.02 Aa	0.59±0.04 Aa	17.95±0.46 Ac	25.00±2.64 Aa	156.33±2.08 Ab	6.92±0.61 Ab	1.19±0.30 Aa
	S1900	7.21±0.14 Aa	1.05±0.07 Aa	33.75±2.91 Aa	54.86±0.86 Aa	2.15±0.03 Ac	0.56±0.01 Aa	29.25±0.43 Aa	17.60±3.48 Bb	248.33±1.53 Aa	10.96±2.37 Aa	1.31±0.06 Aa
	S2100	7.00±0.02 Ab	1.10±0.15 Aa	32.10±1.42 Aab	44.62±0.60 Ab	2.30±0.02 Ab	0.58±0.05 Aa	23.75±1.42 Ab	22.93±1.84 Aab	140.33±2.08 Ac	6.01±0.51 Bb	1.00±0.14 Aa
**North-facing slop**	N1700	7.26±0.12 Aa	1.01±0.04 Aa	32.30±3.20 Ab	34.91±1.72 Aa	2.40±0.03 Aa	0.69±0.06 Aa	18.66±1.42 Ab	21.46±3.12 Ab	129.67±2.08 Bb	5.72±2.31 Ab	0.83±0.52 Aa
	N1900	7.10±0.13 Aab	0.73±0.12 Bb	41.51±1.38 Ba	55.36±1.18 Aa	2.32±0.17 Aa	0.60±0.05 Ab	23.59±1.03 Ba	30.98±1.88 Aa	222.33±1.52 Ba	9.58±0.94 Aa	1.41±0.23 Aa
	N2100	6.95±0.02 Ab	0.97±0.07 Aa	35.21±3.53 Ba	45.15±1.44 Aa	2.25±0.07 Aa	0.54±0.02 Ab	24.50±1.14 Aa	22.92±3.30 Ab	128.33±2.09 Bb	8.67±0.14 Aa	1.15±0.04 Aa
**Slope aspect**		**ns**	**	***	**ns**	**ns**	**ns**	*	**	***	**ns**	**ns**
**Altitude**		**	*	***	***	**	*	***	**ns**	***	**	**ns**
**Slope aspect ×Altitude**		**ns**	**ns**	**ns**	**ns**	*	*	***	***	***	**ns**	**ns**

Note: BD: Soil bulk density; TN: Total nitrogen; TP: total phosphorus; AK: Available potassium; SOC: soil organic carbon; TK: total potassium AP: Available phosphorus; NO_3^--N: Nitrate nitrogen; NH_4^+-N: Ammonium nitrogen; Upper case letters indicate differences between shaded and sunny slopes ($p < 0.05$). Lowercase letters indicate three different elevation gradients ($p < 0.05$). Non-significance is denoted by NS, while *, **, *** indicate statistical significance at $p < 0.05$, $p < 0.01$, $p < 0.001$, respectively. N for north, shady slopes, S for south, sunny slopes, for example: N1700 means 1700m above sea level on the north slope.

Table S2. Two-way variance analysis of dominant bacterial phyla and classes

Taxonomy		Slope aspect		Altitude		Slope aspect×Altitude	
		F	*P*	F	*P*	F	*P*
Phyla	Actinobacteriota	9.505	**	-	-	-	-
	Proteobacteria	-	-	4.705	*	-	-
	Acidobacteriota	-	-	-	-	-	-
	Firmicutes	-	-	15.057	***	-	-
	Chloroflexi	-	-	8.441	**	-	-
	Gemmatimonadota	-	-	7.476	**	-	-
	Verrucomicrobiota	-	-	-	-	-	-
	Methylomirabilota	4.922	*	3.967	*	9.146	**
	Bacteroidota	-	-	8.897	**	-	-
	Myxococcota	5.023	*	-	-	-	-
	Planctomycetota	10.588	**	-	-	-	-
	others	7.771	*	-	-	16.103	***
Classes	Alphaproteobacteria	-	-	4.277	*	-	ns
	Actinobacteria	-	-	7.234	**	4.722	*
	Thermoleophilia	15.128	**	-	-	-	-
	Bacilli	-	-	14.792	***	-	-
	Vicinamibacteria	-	-	-	-	-	-
	Blastocatellia	-	-	-	-	-	-
	Gammaproteobacteria	-	-	-	-	-	-
	KD4-96	-	-	6.199	*	-	-
	Acidimicrobiia	24.658	***	-	-	-	-

MB-A2-108	-	-	10.936	**	-	-
Gemmatimonadetes	-	-	6.593	*	-	-
Verrucomicrobiae	-	-	-	-	-	-
Methylomirabilia	4.922	*	3.967	*	9.146	**
Bacteroidia	-	-	9.360	**	-	-
Chloroflexia	-	-	-	-	-	-
Anaerolineae	-	-	3.970	*	-	-
Acidobacteriae	-	-	-	-	-	-
Gitt-GS-136	-	-	11.087	**	-	-
others	-	-	-	-	7.013	*

Note: - indicates non-significance, *, **, and *** indicate significance at $p < 0.05$, $p < 0.01$ and $p < 0.001$, eachly.

Table S3. Two-way variance analysis of dominant fungal phyla and classes

Taxonomy		Slope aspect		Altitude		Slope aspect×Altitude	
		F	P	F	P	F	P
Phyla	Ascomycota	-	-	-	-	5.727	*
	Mortierellomycota	-	-	-	-	-	-
	Basidiomycota	-	-	-	-	-	-
	unclassified_k__Fungi	-	-	-	-	-	-
	Chytridiomycota	6.805	*	8.110	**	11.330	**
	Glomeromycota	-	-	8.393	**	15.146	***
	others	-	-	-	-	-	-
Classes	Mortierellomycetes	-	-	-	-	-	-
	Sordariomycetes	-	-	17.141	***	4.222	*
	Eurotiomycetes	8.768	*	7.095	**	4.278	*
	Agaricomycetes	-	-	-	-	-	-
	Dothideomycetes	-	-	7.321	**	5.124	*
	Tremellomycetes	-	-	-	-	-	-
	Leotiomycetes	6.127	*	6.639	*	-	-
	unclassified_p_Ascomycota	-	-	-	-	-	-
	unclassified_k_Fungi	-	-	-	-	-	-
	Archaeorhizomycetes	8.522	*	4.669	*	4.338	*
	Chytridiomycota	6.090	*	7.673	**	10.660	**
	Glomeromycetes	-	-	5.098	*	8.594	**
	Pezizomycetes	19.513	***	-	-	-	-
	Microbotryomycetes	-	-	5.363	*	7.586	**
	others	9.102	*	-	-	-	-

Note: - indicates non-significance, *, **, and *** indicate significance at $p < 0.05$, $p < 0.01$ and $p < 0.001$, correspondingly
